# Hydrolytic Capabilities as a Key to Environmental Success: Chitinolytic and Cellulolytic *Acidobacteria* From Acidic Sub-arctic Soils and Boreal Peatlands

**DOI:** 10.3389/fmicb.2018.02775

**Published:** 2018-11-19

**Authors:** Svetlana E. Belova, Nikolai V. Ravin, Timofey A. Pankratov, Andrey L. Rakitin, Anastasia A. Ivanova, Alexey V. Beletsky, Andrey V. Mardanov, Jaap S. Sinninghe Damsté, Svetlana N. Dedysh

**Affiliations:** ^1^Winogradsky Institute of Microbiology, Research Center of Biotechnology of the Russian Academy of Sciences, Moscow, Russia; ^2^Institute of Bioengineering, Research Center of Biotechnology of the Russian Academy of Sciences, Moscow, Russia; ^3^M.V. Lomonosov Moscow State University, Moscow, Russia; ^4^Department of Marine Microbiology and Biogeochemistry, Royal Netherlands Institute for Sea Research, Utrecht University, Den Burg, Netherlands; ^5^Geochemistry, Department of Earth Sciences, Faculty of Geosciences, Utrecht University, Utrecht, Netherlands

**Keywords:** *Acidobacteria*, *Acidisarcina*, lichen-covered tundra, genome annotation, chitinase, chitinolytic ability

## Abstract

Members of the *Acidobacteria* are among the most efficient colonizers of acidic terrestrial habitats but the key traits underlying their environmental fitness remain to be understood. We analyzed indigenous assemblages of *Acidobacteria* in a lichen-covered acidic (pH 4.1) soil of forested tundra dominated by uncultivated members of subdivision 1. An isolate of these bacteria with cells occurring within saccular chambers, strain SBC82^T^, was obtained. The genome of strain SBC82^T^ consists of a 7.11-Mb chromosome and four megaplasmids, and encodes a wide repertoire of enzymes involved in degradation of chitin, cellulose, and xylan. Among those, four secreted chitinases affiliated with the glycoside hydrolase family GH18 were identified. Strain SBC82^T^ utilized amorphous chitin as a source of carbon and nitrogen; the respective enzyme activities were detected in tests with synthetic substrates. Chitinolytic capability was also confirmed for another phylogenetically related acidobacterium isolated from a *Sphagnum* peat bog, strain CCO287. As revealed by metatranscriptomic analysis of chitin-amended peat, 16S rRNA reads from these acidobacteria increased in response to chitin availability. Strains SBC82^T^ and CCO287 were assigned to a novel genus and species, *Acidisarcina polymorpha* gen. nov., sp. nov. Members of this genus colonize acidic soils and peatlands and specialize in degrading complex polysaccharides.

## Introduction

Members of the phylum *Acidobacteria* inhabit a wide variety of terrestrial and aquatic ecosystems (Ludwig et al., 1997; Barns et al., 1999; Kielak et al., 2016; Dedysh and Sinninghe Damsté, 2018; Eichorst et al., 2018). As judged by the proportion of *Acidobacteria*-affiliated 16S rRNA and 16S rRNA gene reads in sequence pools retrieved from different environments, these bacteria are particularly abundant in diverse soil habitats, where they represent between 5 and 50% of the total bacterial community (Janssen, 2006; Lee et al., 2008; Jones et al., 2009; Lauber et al., 2009; Foesel et al., 2014). The highest relative abundances of *Acidobacteria* are commonly observed in acidic soils and peatlands, which are dominated by representatives of subdivisions (Sd) 1 and 3 of this phylum (Jones et al., 2009; Lauber et al., 2009; Serkebaeva et al., 2013).

The reasons behind this high environmental fitness of soil *Acidobacteria* are only partly understood. The first analysis of three genomes from representatives of Sds 1 and 3, i.e., *Acidobacterium capsulatum*, ‘*Koribacter versatilis*’ and ‘*Solibacter usitatus*,’ revealed their potential to participate in the cycling of plant-, fungal-, and insect-derived organic matter (Ward et al., 2009). The genomes encode a wide repertoire of enzymes involved in breakdown and utilization of a diverse suite of carbohydrates. Further genome analyses were also supportive for placing the *Acidobacteria* in the list of organisms involved in hydrolysis and utilization of various biopolymers in nature (Rawat et al., 2012; Kielak et al., 2016). Additional arguments in support of the hydrolytic capabilities of *Acidobacteria* were obtained in a number of cultivation-independent studies. Incubation experiments with ^13^C-labeled cellulose revealed *Acidobacteria* among aerobic decomposers of this biopolymer in an acidic forest soil (Štursová et al., 2012). Several other studies identified members of the family *Acidobacteriaceae* among the anaerobic microorganisms that process cellulose-derived carbon in acidic peatlands (Schmidt et al., 2014; Juottonen et al., 2017). Finally, a specific response to chitin availability was detected for Sd1 *Acidobacteria* in a metatranscriptome-based study with acidic peat (Ivanova et al., 2016).

By contrast, experimental evidence for the hydrolytic capabilities of cultured *Acidobacteria* is rare. Two acidobacterial isolates from agricultural grassland soils obtained by using a complex medium of plant polymeric carbon, strains KBS 83 and KBS 96, were reported to possess cellulolytic potential, but their capability of degrading cellulose was not demonstrated (Eichorst et al., 2011). The presence of a weak chitinolytic capability was first reported for the Sd4 acidobacterium, *Blastocatella fastidiosa* (Foesel et al., 2013) and, later, for two members of Sd1, *Terracidiphilus gabretensis* (García-Fraile et al., 2015) and *Silvibacterium bohemicum* (Lladó et al., 2016). The two latter species were described as possessing cellulolytic potential as well. None of these studies, however, reported related enzyme activities or growth dynamics on cellulose or chitin. Solid experimental evidence for the occurrence of cellulolytic potential was presented only for *Telmatobacter bradus*, which was described as a facultative anaerobe capable of degrading amorphous and crystalline cellulose under micro-oxic and anoxic conditions (Pankratov et al., 2012). The products of cellulose degradation by *Telmatobacter* under anoxic conditions are acetate and hydrogen. Weak cellulolytic capabilities were also demonstrated for a taxonomically uncharacterized Sd1 acidobacterium, strain CCO287, which was isolated from an acidic *Sphagnum* peat bog (Pankratov et al., 2011). This isolate possessed specific cell morphology: cells were highly polymorphic and arranged in cluster-like aggregates. Extremely slow growth in liquid media did not allow investigating hydrolytic capabilities of strain CCO287 in more detail.

In the course of our recent work on the diversity of *Acidobacteria* in lichen-covered soils of forested tundra, we obtained another, phylogenetically related isolate with the same specific morphology as strain CCO287. It showed a more consistent growth allowing a detailed characterization of its physiology and specific adaptations. We also analyzed its genome-encoded traits and hydrolytic capabilities, which reveal the reasons behind wide distribution of these *Acidobacteria* in acidic soils and peatlands.

## Materials and Methods

### Study Site and Sampling Procedure

**FIGURE 1:**
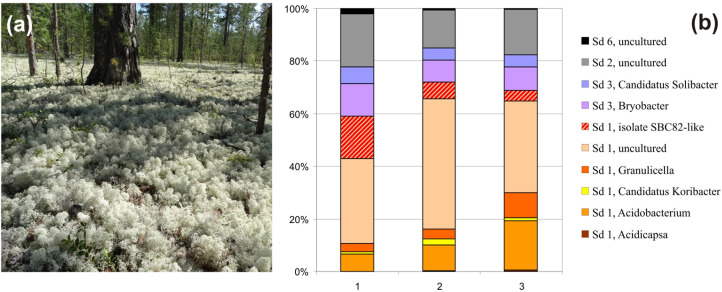
**(a)** The forested tundra site examined in this study: pine forest with ground vegetation cover composed of reindeer lichens. **(b)** Community composition of the *Acidobacteria* within three individual plots of the forested tundra based on Illumina paired-end sequencing of 16S rRNA gene fragments. The proportion of reads assigned to isolate SBS82-like bacteria was determined based on the sequence identity threshold of 97%.

The study site was located in the Nadym region of northwest Siberia, Yamalo-Nenets AO, Russia, in a pine (*Pinus sibirica*) forest within a permafrost-free zone of forested tundra (N65°36′07.1″, E72°44′39.5″). The ground vegetation was composed of lichens (*Cladonia stellaris*, *Cladonia alpestris*, and *Cetraria islandica*) with a minor presence of *Vaccinium* spp., *Ledum palustre*, and *Polytrichum commune* (Figure 1a). Three individual plots, at a distance of 15–20 m from each other, were chosen for sampling purposes. The soil samples (ca. 500 g each) were collected from the surface (1–5 cm depth) organic soil layer (pH 4.1) underlying the lichen cover. The samples were transported to the laboratory in boxes containing ice packs, homogenized, and frozen at −20°C for DNA extraction within 1 day after sampling.

### High-Throughput Sequencing of 16S rRNA Genes

Three individual soil samples obtained from three different plots, each of 0.5 g wet weight, were taken for the analysis and processed separately. Isolation of total DNA from soil samples was performed using FastDNA SPIN kit for soil (MP Biomedicals, United States) and FastPrep-24 homogenizer (MP Biomedicals, United States) in accordance with manufacturer’s instructions. Subsequent purification of DNA samples was performed using preparative gel electrophoresis and Cleanup Standard Kit (Evrogen, Russia). The V4 region of 16S rRNA genes was amplified from the DNA samples using the 515f/806r primer set (Caporaso J.G. et al., 2010) with some primer modifications: as forward primer the Univ515F primer was used (5′-GTG BCA GCM GCC GCG GTA A-3′; Kublanov et al., 2009). The resulting amplicons were purified by agarose gel electrophoresis using Cleanup Standard Kit (Evrogen, Russia) and sequenced using the 300PE protocol on MiSeq System (Illumina, United States). Resulting reads were subjected to stringent quality filtering and trimming with CLC Genomics Workbench 7.5 (Qiagen, Germany). After filtering, overlapping paired-end library reads were merged with SeqPrep tool^1^. Demultiplexing and further processing of the resulting data set was carried out using QIIME v.1.8. package (Caporaso J. et al., 2010). Taxonomic assignment was performed using RDP classifier retrained with Silva 128 database (Pruesse et al., 2007; Quast et al., 2013). Culling of chimeric sequences was performed using ChimeraSlayer algorithm (Grabherr et al., 2011).

### Cultivation Studies

Ten grams of a surface soil enriched with decomposed thalli of *Cladonia* sp. were grinded in a mortar and the resulting material was suspended in 50 ml of sterile water. This suspension was further diluted (1:50) and spread-plated onto the medium solidified with 9 g phytagel (Sigma-Aldrich) and containing (per liter distilled water): 0.25 g xylan, 0.25 g starch, 0.05 g Bacto Tryptic Soy Broth without Dextrose, 0.1 g NH_4_NO_3_, 0.1 g MgSO_4_⋅7H_2_O, 0.04 g KH_2_PO_4_; pH was adjusted to 4.5 with 30 mg alginic acid l^−1^. The plates were incubated at 20°C for 4 weeks. Colonies that developed on these plates were screened using the *Acidobacteria*-specific PCR approach (Stevenson et al., 2004). One particular type of colonies that were identified as belonging to members of the *Acidobacteria* was represented by small (1–3 mm in diameter after 4 weeks of incubation), gummy consistency, opal-beige to light pink in color, circular colonies. Cell material from these colonies was further re-streaked on the above described medium and the plates were incubated under the same conditions until the target bacterium, designated strain SBC82^T^, was obtained as a pure culture. Strain SBC82^T^ was maintained on the solid medium MA containing (per liter distilled water): 0.5 g fructose, 0.1 g yeast extract, 0.04 g MgSO_4_⋅7H_2_O, 0.04 g KH_2_PO_4_, 0.02 g CaCl_2_⋅2H_2_O, 0.2 g KNO_3_, 9 g phytagel, 0.03 g alginic acid, pH 4.6–5.0 and was sub-cultured at 3-week-intervals.

The isolation and brief characterization of strain CCO287 was reported earlier (Pankratov et al., 2011). This bacterium was isolated from *Sphagnum*-derived peat collected from the ombrotrophic peat bog Obukhovskoye, Yaroslavl region, European North Russia (58° 14′N, 38° 12′E) by means of plating onto an acidic (pH 4.5–5.0) medium with cellulose powder (Aldrich). Strain CCO287 was maintained on the medium MA supplied with 2 ml vitamin solution of the following composition (g per 100 ml distilled water): p-aminobenzoate, 1.0; biotin, 0.2; nicotinic acid, 2.0; thiamine-HCl× 2H_2_O, 1.0; Ca-pantothenate, 0.5; pyridoxamine, 5.0; vitamin B12, 2.0.

### Microscopy

Morphological observations and cell size measurements were made with a Zeiss Axioplan 2 microscope and Axiovision 4.2 software (Zeiss, Germany). For preparation of ultrathin sections, cells of the exponentially growing culture were collected by centrifugation and pre-fixed with 1.5% (w/v) glutaraldehyde in 0.05 M cacodylate buffer (pH 6.5) for 1 h at 4°C and then fixed with 1% (w/v) OsO_4_ in the same buffer for 4 h at 20°C. After dehydration in an ethanol series, the samples were embedded into Epon 812 epoxy resin. Thin sections were cut on an LKB-4800 microtome, stained with 3% (w/v) uranyl acetate in 70% (v/v) ethanol, and then were stained with lead citrate (Reynolds, 1963) at 20°C for 4–5 min. The specimen samples were examined with JEM-1011 (JEOL, Japan) transmission electron microscope.

### Chemotaxonomic Analyses

For the analysis of total lipids (including fatty acids), cells of strains SBC82^T^ and CCO287 were grown in liquid medium MA and harvested in the late exponential growth phase. Lipids were analyzed after acid hydrolysis of the cell material following the procedure described by Sinninghe Damsté et al. (2011). The intact polar lipids (IPLs) in these bacteria were analyzed as described by Moore et al. (2013).

Isoprenoid quinones were extracted according to Collins (1985) and analyzed using a tandem-type mass spectrometer LCQ ADVANTAGE MAX and a Finnigan Mat 8430 ionization mass spectrometer with atmospheric pressure chemical ionization (APCI).

### Phenotypic Characterization

Physiological tests were performed using batch cultures grown in the liquid medium MA without phytagel. Growth of strain SBC82^T^ was monitored by nephelometry at 410 nm using “Specol” spectrophotometer (Carl Zeiss) for 14 days under a variety of conditions, including temperatures of 2–37°C, pH 3.0–7.9 and NaCl concentrations of 0–4.0% (w/v). Incubations at various temperatures were made under static conditions in triplicate. Variations in pH were achieved by using MES (pH 4.0–6.5) and MOPS (pH 6.5–7.9) buffer systems. The pH range of 3–4 was achieved by adjusting the medium pH with 0.1 M H_2_SO_4_. The range of potential growth substrates of strain SBC82^T^ was examined on liquid medium MA by replacing fructose with respective carbon sources (0.05% [w/v]) and decreasing the concentration of yeast extract to 0.005% (w/v). Cultivation was done in 30 ml vials containing 5 ml medium. The inoculated cells were washed with a fresh medium without any substrate before a new batch culture was inoculated. The incubations were kept at 22°C for 3 weeks on a shaker. All experiments were performed in triplicate. Enzymatic profiles, urease, β-galactosidase activity, indole production, abilities to hydrolyze gelatin and esculin were examined with API ZYM and API 20NE kits (bioMérieux). A catalase test was carried out by using the standard method (Gerhardt, 1981). Oxidase was tested using a REF 55 635 Oxidase Reagent (bioMérieux). The ability to grow under anaerobic conditions was tested in anaerobic jars by using AnaeroGen anaerobic system envelopes (Oxoid), which absorb atmospheric oxygen with the simultaneous generation of CO_2_ (up to 9–13%, vol/vol).

### Growth Experiments and Whole-Cell Hybridization

Strain SBC82^T^ was grown under static conditions at 25°C in 160 ml serum bottles containing 20 ml of liquid medium MA without fructose. Amorphous chitin was prepared as described elsewhere (Sorokin et al., 2014) and added to the medium at a concentration of 0.1% (w/v). In one set of experimental flasks, chitin was provided as a sole source of carbon, while KNO_3_ (0.02%, w/v) was supplied as a chitin-independent source of nitrogen. In the second set of incubation flasks, chitin was added as the sole source of carbon and nitrogen, while KNO_3_ was omitted from the medium composition. Control incubations without chitin were run in parallel under the same conditions. All incubations were performed in triplicate. After 20 days of incubation, the culture suspensions were fixed with 4% (wt/vol) freshly prepared paraformaldehyde solution as described by Dedysh et al. (2001). The *Acidobacteria*-specific Cy3-labeled oligonucleotide probe HoAc1402 (5′-CTTTCGTGATGTGACGGG-3′) (Juretschko et al., 2002) was applied for specific detection of cells on micro-particles of chitin. The oligonucleotide probe was purchased from Syntol (Moscow, Russia). Hybridization was done on gelatin-coated (0.1%, wt/vol) and dried Teflon-laminated slides (MAGV, Germany) with eight wells for independent positioning of the samples. The fixed samples were applied to these wells, hybridized to the corresponding fluorescent probes, and stained with the universal DNA stain 4′,6-diamidino-2-phenylindole (DAPI, 1 μM). Cell counts were carried out with a Zeiss Axioplan 2 microscope (Zeiss, Jena, Germany) equipped with the Zeiss Filters No 20 and 02 for Cy3-labeled probes and DAPI staining, respectively. Cell counting was performed on 100 randomly chosen fields of view (FOV) for each test sample. The number of target cells per ml of culture suspension was determined from the area of the sample spot, the FOV area, and the volume of the fixed aliquot used for hybridization.

### Hydrolytic Activity Assays

Cells of strain SBC82^T^ grown in the liquid medium MA supplemented with 0.1% (w/v) amorphous chitin instead of fructose were collected by centrifugation and lysed by sonication. Chitinolytic activities of the resulting cell extracts were measured in a fluorimetric assay with 4-methylumbelliferyl (4-MU) derivatives using a Chitinase Assay Kit (Sigma, CS1030). The following substrates were used: 4-MU- β-D-N, N′, N″-triacetylchitotriose, 4-MU-diacetyl-β-D-chitobioside and 4-MU-*N*-acetyl-β-D-glucosaminide. One unit of activity was defined as the amount of enzyme required to release 1 nmol of 4-MU from the appropriate substrate per minute. Assays were performed at 25°C in 50 mM sodium citrate buffer (pH 5.0). The fluorescence of liberated 4MU was measured using the fluorimeter Fluorat-02-Panorama (Lumex, Russian Federation), with excitation at 360 nm and emission at 450 nm.

### Genome Sequencing, Annotation, and Analysis

For genome analysis, strain SBC82^T^ was grown in shaking liquid cultures at 25°C in medium MA. After 3 weeks of incubation, the biomass was collected and used for DNA extraction, library preparation, and sequencing. Genomic DNA of strain SBC82^T^ was sequenced with a Roche Genome Sequencer (GS FLX), using the Titanium XL+ protocol for a shotgun genome library and MinION (Oxford Nanopore), using 1D Genomic DNA by ligation protocol. About 244 Mb of sequences with an average read length of 549 nt were generated on GS FLX and *de novo* assembled into contigs using Newbler Assembler version 2.9 (454 Life Sciences, Branford, CT, United States). 117573 raw MinION reads (about 1.1 Gb in total) were corrected and trimmed using Canu assembler (Koren et al., 2017). Corrected MinION reads were used to finish draft GS FLX *de novo* assembly with npScarf software (Cao et al., 2017). GS FLX reads were mapped back to the finished sequence using Bowtie 2 (Langmead and Salzberg, 2012) and the mapping was used to calculate improved consensus sequence by Pilon software (Walker et al., 2014).

Gene search and annotation were performed using the RAST server (Brettin et al., 2015), followed by manual correction by searching the National Center for Biotechnology Information (NCBI) databases. Signal peptides were predicted using Signal P v.4.1 for Gram-negative bacteria^2^. The N-terminal twin-arginine translocation (Tat) signal peptides were predicted using PRED-TAT^3^ and the transmembrane helices with TMHMM Server v. 2.0^4^. The values of DNA–DNA hybridization *in silico* were calculated using GGDC 2 (Meier-Kolthoff et al., 2013), available at http://ggdc.dsmz.de/, according to formula 1.

### Analysis of Metatranscriptome-Derived Data

The SSU rRNA dataset retrieved in the study of Ivanova et al. (2016) was used to analyze the abundance of *Acidisarcina polymorpha-*like acidobacteria in *Sphagnum*-derived peat and the substrate-induced response of these bacteria to amendments with different biopolymers. The 16S rRNA gene sequences of *A. polymorpha* strains SBC82^T^ and CCO287 were used as queries for blastn search via *blast+* (Camacho et al., 2009) among the SSU rRNA reads with an identity thresholds of 95 and 99%. One-way ANOVA followed by Dunnett’s multiple comparisons test (i.e., comparison of the results obtained for biopolymer-amended samples with those of the control samples) was performed using GraphPad Prism version 6.00 for Windows, GraphPad Software, La Jolla, CA, United States ^5^. Tests were considered significant if they had a *P*-value < 0.05.

### Sequence Accession Numbers

The 16S rRNA gene reads retrieved using Illumina paired-end sequencing from the forested tundra soil samples (raw data) have been deposited under the Bioproject number PRJNA344855 in the NCBI Sequence Read Archive, with the accession number SAMN05846486. The annotated genome sequence of strain SBC82^T^ has been deposited in GenBank under the accession numbers CP030840-CP030844. The GenBank/EMBL/DDBJ accession numbers for the 16S rRNA gene sequences of strains SBC82^T^ and CCO287 are MH396772 and FR750264, respectively.

## Results

### Acidobacteria Diversity in an Acidic Tundra Soil

A total of 682,752 partial 16S rRNA gene sequences (mean amplicon length 253 bp) were retrieved from the three examined soil samples. Of these, 606,366 reads were retained after quality filtering and denoising of the raw data. Only a minor part of these sequences (0.1–0.4%) were classified as belonging to the *Archaea*, more specifically to uncultivated members of the *Thaumarchaeota*. Major groups of bacteria in lichen covered tundra soil were represented by the *Acidobacteria* (22–24% of all classified 16S rRNA gene sequences), *Proteobacteria* (33–40%), *Planctomycetes* (13–16%), *Actinobacteria* (11–13%), and *Verrucomicrobia* (9–10%).

The pool of 16S rRNA gene fragments assigned to the *Acidobacteria* included 157,407 reads. Nearly equal numbers of reads (29,264 and 36,224) were obtained from samples No 1 and 3. The number of reads retrieved from sample No 2 was much larger than the others (91,919 reads) and, therefore, ∼34,000 reads were randomly selected from this sequence pool for further analysis (Table 1). The final set of 16S rRNA gene fragments from the *Acidobacteria* used for the analysis included 99,488 reads. The calculated Chao1 and Shannon indexes for individual samples were in the range of 837–1,014 and 5.3–5.6, respectively. The number of species-level OTUs determined at 97% sequence identity ranged between 550 and 685.

**Table 1:** Sequencing statistics and various alpha-diversity metrics for the subset of 16S rRNA gene fragments assigned to the *Acidobacteria.*

Sample No	Raw reads	Filtered reads^∗^	Reads from *Acidobacteria*	Diversity indices				
				Chao1	Shannon	Observed species^∗∗^	Menhinick	Good’s coverage estimator
1	128,572	112,642	29264	837.8	5.4	550	3.2	0.992
2	402,892	356,887	91919 (34000)	1070.8 (1014.7)	5.6 (5.6)	974 (685)	3.2 (3.7)	0.997 (0.992)
3	151,288	136,837	36224	855.2	5.3	612	3.2	0.993

*^∗^Filtered reads: number of sequences excluding singletons and chimera sequences. ^∗∗^Calculations are based on a sequence similarity cut-off of 97%. The randomized number of sequences is given in parentheses for the overrepresented sample 2.*

Taxonomic analysis revealed that the surface soil in lichen covered tundra was dominated by Sd1 *Acidobacteria* (59–71% of total *Acidobacteria*-like reads), while Sd2, Sd3 and, to a much lesser extent, Sd6 were also present (14–20, 13–19, and 0–2%, respectively) (Figure 1b). Uncultivated members of the family *Acidobacteriaceae* were the major fraction of Sd1 *Acidobacteria* in these soils (32–49% of total *Acidobacteria*-like reads). The sequences affiliated with the genera *Acidobacterium* and *Granulicella* comprised two major groups of taxonomically described Sd1 *Acidobacteria* (7–18 and 3–9% of total reads, respectively).

### Cultivation Studies

**FIGURE 2:**
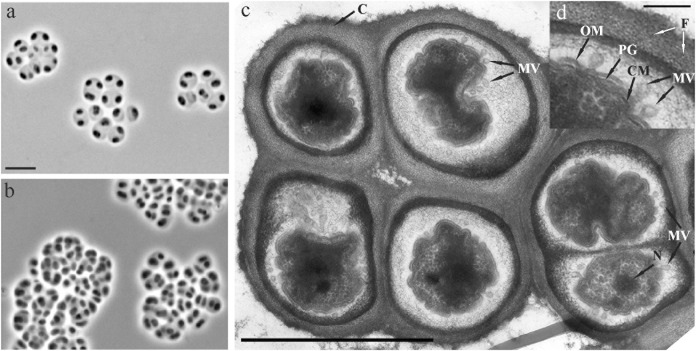
**(a,b)** Phase-contrast images of cells of strain SBC82^T^ grown for 7 days **(a)** and 2 weeks **(b)** in the liquid medium MA supplemented with glucose **(a)** and xylan **(b)** as carbon sources. **(c)** Electron micrograph of an ultrathin section of a cell aggregate from a glucose-grown culture. **(d)** Enlarged view of outer membrane vesicles formed by invaginations of cytoplasmic membrane. CM, cytoplasmic membrane; OM, outer membrane; PG, peptidoglycan layer; MV, membrane vesicles; N, nucleoid; C, saccular chambers; F, fibril-like structures. Bars, 5 μm **(a,b)**, 1 μm **(c)**, and 0.1 μm **(d)**.

**FIGURE 3:**
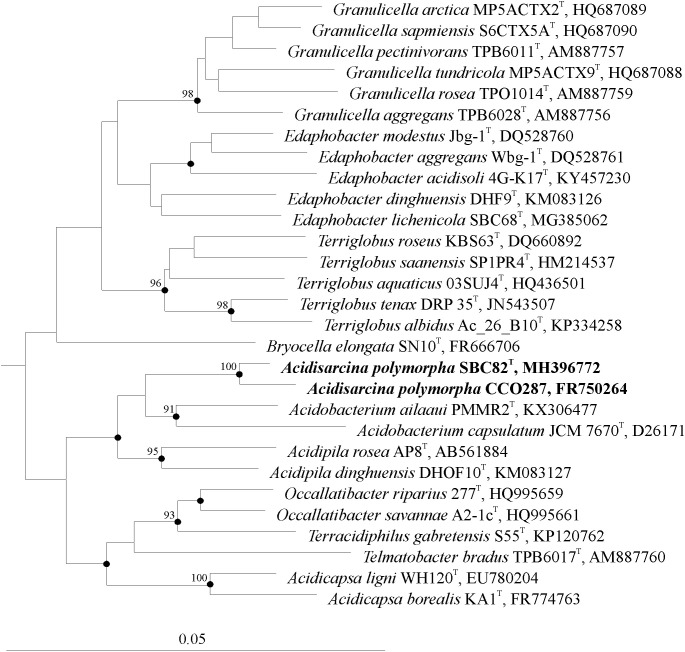
Unrooted 16S rRNA gene-based neighbor-joining tree showing the phylogenetic relationship of strains SBC82^T^ and CCO287 to representative members of the family *Acidobacteriaceae.* The tree was calculated based on 1,380 nt positions using the ARB program package (Ludwig et al., 2004). The significance levels of interior branch points obtained in the neighbor-joining analysis were determined by bootstrap analysis (based on 1,000 data resamplings) using PHYLIP (Felsenstein, 1989). Bootstrap values of >90% are shown. Black circles indicate that the corresponding nodes were also recovered in the maximum-likelihood and maximum-parsimony trees. Bar, 0.05 substitutions per nucleotide position.

About one hundred of colonies that developed on the acidic (pH 4.5) phytagel-solidified medium with xylan and starch as carbon sources were screened by using the *Acidobacteria*-specific PCR. Of those, 37 colonies were identified as belonging to members of the phylum *Acidobacteria* and selected for further analysis. Most of these colonies had a slimy consistency, were light pink to pink in color and were composed of rod-shaped cells. Identification of these isolates by partial (∼600 bp) 16S rRNA gene sequencing revealed that they belonged to the genera *Granulicella* (24 isolates)*, Terriglobus* (1 isolate), *Edaphobacter* (2 isolates), and *Acidicapsa* (3 isolates), and displayed 97–99% 16S rRNA gene similarity to described species of these genera. Seven morphologically distinct colonies had gummy consistency, were opal-beige to light pink in color and were composed of polymorphic cells that were arranged in sarcina- or cluster-like aggregates (Figure 2a). This cell morphology was highly similar to that of our earlier described acidobacterial isolate CCO287 from an acidic *Sphagnum* peat bog (Pankratov et al., 2011). A representative isolate of these bacteria from the lichen-covered tundra soil, strain SBC82^T^, was obtained in a pure culture and was selected for further study. The 16S rRNA gene sequence of strain SBC82^T^ was highly similar (99.1%) to that of strain CCO287 but displayed only 92–96% similarity to 16S rRNA gene sequences from other currently described members of the family *Acidobacteriaceae* (Figure 3). The closest phylogenetic relatives of strains SBC82^T^ and CCO287 were representatives of the genera *Acidobacterium*, *Telmatobacter*, *Occallatibacter*, *Terracidiphilus*, *Acidicapsa*, and *Acidipila*.

### Cell Morphology

Strains SBC82^T^ and CCO287 were non-spore-forming, highly polymorphic bacteria that, most commonly, occurred in pairs, in sarcina-like tetrads, and in clusters of at least six to eight cells (Figures 2a,b). Formation of large and tightly packed cell clusters was observed during growth on polymeric substrates, like xylan (Figure 2b). Single cells or short chains of curved cells could also be observed occasionally. Old cultures contained strongly aggregated cells. Cells were 1.4–4.4 μm long and 0.9–1.5 μm wide. Electron microscopy revealed a cell-wall structure typical of Gram-negative bacteria. The cytoplasmic membrane, peptidoglycan layer and outer membrane were evident in ultrathin sections (Figure 2c). The specific feature of cell morphology of these bacteria was the presence of extracellular structures appearing as saccular chambers of a complex ultrastructure. The chamber envelope was structurally organized by tightly packed fibrils layered in parallel to each other (Figure 2d). This envelope could have been originated from numerous outer membrane vesicles, which were produced by cells, released in the chamber matrix and transferred to the inner surface of the chamber envelope.

### Chemotaxonomic Characterization

In order to taxonomically characterize the two acidobacterial isolates, we analyzed their fatty acid and polar lipid composition. The fatty acid patterns obtained for strains SBC82^T^ and CCO287 were highly similar and were dominated by 13,16-dimethyl octacosanedioic acid (29.8–30.7%), iso-C15:0 (24.6–29.4%), C16:1ω7*c* (18.5–20.4%), and C16:0 (8.1–10.3%) (Table 2). High amounts of these fatty acids are commonly found in members of the family *Acidobacteriaceae* (Sinninghe Damsté et al., 2011). The IPLs in both strains SBC82^T^ and CCO287 were represented by phosphatidylethanolamine (PE), phosphohexose (P-hex) and smaller amounts of an unknown high mass IPL (Table 3). Strain CCO287 contained ornithine lipid (OL) as a major component, while it was present only as a minor component in IPL profile of SBC82^T^. Similar to all currently described members of the family *Acidobacteriaceae*, strains SBC82^T^ and CCO287 contained menaquinone-8 (MK-8) as the predominant isoprenoid quinone.

**Table 2:** Fatty acid composition (%) released after acid hydrolysis of cell material of strains SBC82^T^ and CCO287.

Fatty acids	SBC82^T^	CCO287
C14:0	0.4	0.4
iso-C15:0	**29.4**	**24.6**
C16:1ω7*c*	**20.4**	**18.5**
C16:1ω7*t*	0.2	1.1
C16:0	**8.1**	**10.3**
iso-C17:1ω7	2.5	2.3
iso-C17:0	1.5	2.4
ai-C17:0	0.7	0.5
C17:1ω8	0.6	0.3
C18:2	–	0.4
C18:1ω9	2.8	**5.8**
C18:0	0.9	1.2
C20:0	0.2	0.6
C22:1ω9	–	0.3
C22:0	1.6	1.3
13,16-Dimethyl octacosanedioic (iso-diabolic acid)	**30.7**	**29.8**

*Major components (>5%) are given in bold type. –, not detected.*

**Table 3:** Relative abundances ^a^ of intact polar lipids of strains SBC82^T^ and CCO287.

IPL^b^	SBC82^T^	CCO287
Unknown IPL (m/z 751)	++	+
PE	+ + +	+ + +
OL	+	+ + +
P-hex	++	++

*^a^The abundance is relative to major peak in the LC/MS base peak chromatogram: + + +, base peak; ++, 50–100% of base peak; +, 10–50% of base peak. Note that the mass spectral response factors for different IPL groups can be quite different. ^b^Listed in order of elution: PE, phosphatidyl ethanolamine; OL, ornithine lipid; P-hex, phosphohexose.*

### Physiology of Isolates

Strains SBC82^T^ and CCO287 were aerobic, acidophilic and psychrotolerant chemoheterotrophs, which grew at pH values between 4.0 and 7.7 (optimum pH 4.8–7.0) and at temperatures between 5 and 36°C (optimum at 20–32°C). Sugars and some polysaccharides, like starch and xylan, were the preferred growth substrates. By contrast to strain SBC82^T^, strain CCO287 required supplementation with vitamins (with biotin as a key component) for growth. The complete list of carbon sources utilized by these bacteria and the enzyme activities revealed by the API ZYM test are listed in the species description (see below). Strain SBC82^T^ and CCO287 showed poor growth in micro-oxic conditions but fermentation products were not detected. Growth under strictly anaerobic conditions was not observed. NaCl inhibited growth at concentrations above 1.5% (wt/vol).

### Growth on Chitin and Chitinolytic Activities

**FIGURE 4:**
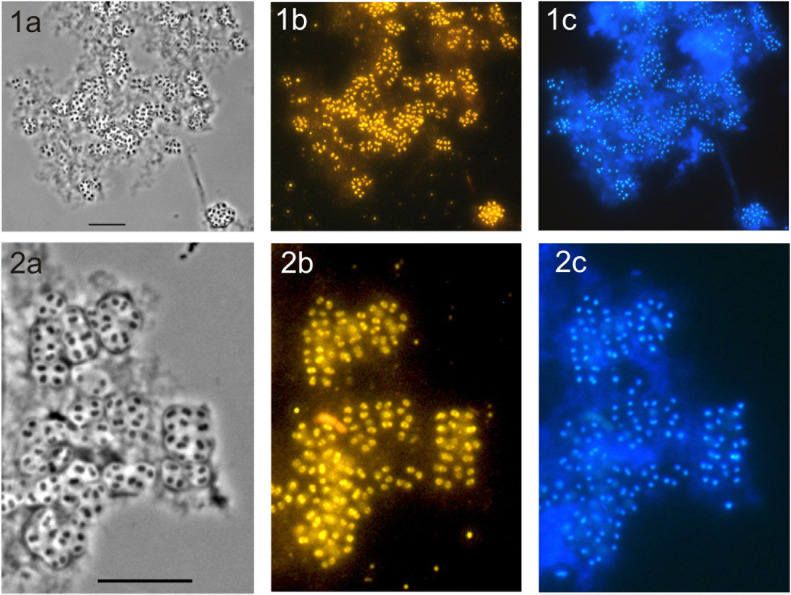
Specific detection of cells of strain SBC82^T^ on micro-particles of amorphous chitin used in growth experiments as a sole source of carbon and nitrogen. The cultures were pictured after 20 days of incubation. Phase-contrast images **(a)**, the respective epifluorescent micrographs of whole-cell hybridizations with Cy3-labeled probe HoAc1402 **(b)**, and DAPI-staining **(c)** are shown. Rows (**1**) and (**2**) demonstrate different fields of view. Enlarged images in row (**2**) demonstrate characteristic cellular aggregates of strain SBC82^T^. Bars, 10 μm.

Given a clearly pronounced response to chitin availability observed for subdivision 1 *Acidobacteria* in our recent metatranscriptome-based study with acidic peat (Ivanova et al., 2016), we tested the ability of novel isolates to grow on this biopolymer. Since strain CCO287 did not grow well in liquid media, detailed experiments on chitin utilization were performed with strain SBC82^T^. In these tests, chitin was provided either as a sole source of carbon and nitrogen or nitrate was supplemented in addition to chitin to provide an alternative source of nitrogen. Strain SBC82^T^ demonstrated good growth in both types of incubations, although it grew slightly better in the presence of mineral nitrogen (Table 4). Microscopic examination of the respective cultures after 3 weeks of incubation revealed numerous cells of strain SBC82^T^ being attached to micro-particles of amorphous chitin (Figure 4). Cells were highly aggregated and occurred within well-visible saccular chambers (images 2a-2c in Figure 4). No free-floating cells were observed.

**Table 4:** Cell numbers of SBC82^T^ determined by whole-cell hybridizations with Cy3-labeled probe HoAc1402 after 20 days of incubation with chitin as a source of carbon and nitrogen or as a source of carbon.

Experiment	Incubation with chitin		Incubation with chitin and ${\text{NO}}_{3}^{-}$	
	+chitin	-chitin	+chitin, $+{\text{NO}}_{3}^{-}$	-chitin, $+{\text{NO}}_{3}^{-}$
I	182.7 ± 34.9	0.3 ± 0.1	228.8 ± 33.4	0.4 ± 0.3
II	238.2 ± 25.5	0.4 ± 0.3	305.8 ± 35.3	0.7 ± 0.4
III	164.9 ± 28.8	0.3 ± 0.1	214.9 ± 29.0	0.5 ± 0.2

*The results of three independent incubations (I–III) are shown. Values are numbers of cells ml^−1^ (N ± standard errors × 10^6^).*

Chitinolytic activities of strain SBC82^T^ grown in chitin-containing medium were detected in cell extracts. Activity was maximal with β-*N*-acetylglucosaminidase substrate 4-MU-*N*-acetyl-β-D-glucosaminide (32.0 × 10^−9^ U cell^−1^), while about 16-fold lower activity was observed with 4-MU-diacetyl-β-D-chitobioside (2.0 × 10^−9^ U cell^−1^) and about 100-times lower activity was recorded with the endochitinase substrate 4-MU-β-D-*N*, *N*′, *N*″-triacetylchitotriose (0.32 × 10^−9^ U cell^−1^).

The tests for the presence of chitinolytic capability in strain CCO287 were also positive although only a slow growth was observed. This bacterium was shown to be capable of slow growth on fibrous cellulose in our former study (Pankratov et al., 2011). Strain SBC82^T^ was also tested for growth on cellulose but only a weak growth was recorded. The two isolates differed also with regard to pectin utilization, which was positive for strain CCO287 but negative for strain SBC82^T^. Apparently, these two closely related acidobacteria displayed some clear difference with regard to their hydrolytic capabilities.

### Genome Characterization

To reveal the genetic background of chitinolytic capability, the complete genome sequence was obtained for strain SBC82^T^. It consists of a circular 7,112,011 bp long chromosome with a GC content of 56.8%, and four circular plasmids with lengths of 62,450 bp, 108,812 bp, 196,405 bp, and 121,048 bp. A single 16S-23S-5S rRNA operon and 46 tRNA genes were identified. Gene calling and annotation of the genome predicted 7,268 potential protein-coding genes of which 4,227 (58%) could be functionally assigned.

The value of *in silico* DNA–DNA hybridization between strain SBC82^T^ and its closest relatives (Figure 3) with determined genome sequences, *Acidobacterium ailaaui* PMMR2^T^ and *Acidobacterium capsulatum* ATCC 51196^T^, was estimated to be about 13%, indicating that strain SBC82^T^ is phylogenetically distant from the closest genus *Acidobacterium*.

Genome analysis revealed a set of genes encoding a bacterial flagellar apparatus and chemotaxis functions, suggesting that cells of strain SBC82^T^ could be motile under certain growth conditions. The cellulose synthase operon encoding the endoglucanase BcsZ, subunit BcsQ, the main catalytic glycosyltransferase subunit BcsA fused to the cyclic di-GMP binding subunit BcsB, and the periplasmic subunit BcsC were revealed in the genome of SBC82^T^.

Analysis of metabolic pathways revealed that this bacterium is metabolically versatile. The genome of SBC82^T^ contains a complete set of genes encoding enzymes of Embden-Meyerhof and pentose phosphate pathways of carbohydrate metabolism, and the tricarboxylic acids cycle. The pyruvate produced in the glycolysis could be oxidized by pyruvate-flavodoxin oxidoreductase, followed by conversion of acetyl-CoA to acetate with the concomitant production of ATP via a two-step reaction by phosphate acetyltransferase and acetate kinase. Genes for the fermentative production of ethanol and lactate were also present. Cytoplasmic [NiFe] Group 3d: hydrogenase HoxEFUYH could catalyze fermentative NADH-dependent production of H_2_.

Glycogen may be used as a storage polysaccharide as suggested by the presence of genes coding for the key enzymes involved in its synthesis (glucose-1-phosphate adenylyltransferase, glycogen synthase and glycogen branching enzyme). Enzymes for two pathways in the synthesis of another storage carbohydrate, trehalose, were encoded: trehalose synthase, and a pair of maltooligosyl trehalose synthase and maltooligosyl trehalose trehalohydrolase.

Consistently with an aerobic lifestyle of strain SBC82^T^, the major electron transport chain was found, namely NADH ubiquinone oxidoreductase, membrane-linked succinate dehydrogenase, cytochrome *bc_1_* complex, cytochrome *c* oxidase of the *caa_3_* type, and an F-type H^+^-transporting ATPase. Pathways for dissimilatory reduction of sulfate, Fe(III), nitrate and nitrite were not found. However, strain SBC82^T^ potentially has capacities for anaerobic respiration since its genome encodes three membrane bound molybdopterin oxidoreductases, but specificity of these complexes could not be reliably predicted from the sequence data. The list of genes discussed above is given in Supplementary Table S1.

The search for potential hydrolytic enzymes revealed >100 glycoside hydrolases, as well as a wide range of other enzymes involved in transport and metabolism of carbohydrates. The extracellular hydrolysis of chitin could be performed by concerted action of chitinases that hydrolyze chitin to oligo- and disaccharides, and *N*-acetyl-beta-hexosaminidase which further degrades chitooligomers into *N*-acetylglucosamine (GlcNAc) monomers (Hunt et al., 2008). Genome analysis revealed four endochitinases of GH18 family and three GH20 family *N*-acetyl-beta-hexosaminidases, predicted to contain the N-terminal secretion signals suggesting their extracellular operation (Supplementary Table S2). Transport of GlcNAc and its oligomers across the outer and cytoplasmic membranes could be enabled by carbohydrate-selective porins (Li and Roseman, 2004) and *N*-acetylglucosamine-related transporters (NagX), respectively. Two intracellular GH3 family hexosaminidases and one GH18 family chitinase lacking recognizable N-terminal signal peptide could be responsible for further hydrolysis of short GlcNAc oligomers.

In the cytoplasm, GlcNAc may be phosphorylated to yield GlcNAc-6-P by N-acetylglucosamine kinase. Upon deamination and deacetylation GlcNAc-6-P is converted into fructose-6-P that enters Embden-Meyerhof pathway (Beier and Bertilsson, 2013), while ammonium could be used as a nitrogen source. Similar to truly chitinolytic bacteria such as *Chitinispirillum alkaliphilum* (Sorokin et al., 2014), the genome of strain SBC82^T^ also encodes enzymes responsible for intracellular metabolism of GlcNAc dimers, i.e., chitobiose phosphorylase of GH36 family that can generate GlcNAc and GlcNAc-1-P, and acetylglucosamine-1-P-mutase making GlcNAc-6-P from GlcNAc-1-P.

According to the growth experiments, SBC82^T^ was capable of growth on starch, xylan, and cellulose; the corresponding pathways were also found in the genome (Supplementary Table S2). The presence of four extracellular and five intracellular enzymes related to alpha amylases could enable utilization of starch. The presence of two extracellular endo-1,4-β-xylanases of GH10 family and three beta-xylosidases is consistent with the ability to grow on xylans. Several signal peptide-containing glycosyl hydrolases could be responsible for extracellular hydrolysis of cellulose. Genome analysis revealed three cellulases of GH5 family, a GH44 family endo-1,4-β-glucanase, and six β-glucosidases. The signal peptide-containing GH16 family glycosyl hydrolase could hydrolyse 1,3-β-glucosidic linkages in 1,3-β-D-glucans such as laminarin, consistently with the observed growth of strain SBC82^T^ on this substrate. The GH64 family enzyme could be also responsible for extracellular hydrolysis of 1,3-β-D-glucans.

Although growth on lactate was not observed, the genome of strain SBC82^T^ contains two lactate utilization *lutABC* operons encoding lactate dehydrogenase (Supplementary Table S1). This membrane-associated complex is responsible for oxidation of L-lactate to pyruvate in *Bacillus subtilis* (Chai et al., 2009) and is widespread in bacteria; in SBC82^T^ this enzyme could drive the oxidation of lactate under aerobic conditions. Another trait that could contribute to adaptation of SBC82^T^ to nutrient limitation is the [NiFe] group 1h hydrogenase. Such unidirectional uptake hydrogenases have high affinity and enable scavenging electrons from atmospheric H_2_ to fuel aerobic respiratory chain when organic electron donors are scarce (Greening et al., 2015).

### Metatranscriptome-Based Evidence for the Presence of Hydrolytic Potential

Since one of the isolates described in this study, strain CCO287, was obtained from a *Sphagnum* peat used in our recent metatranscriptomic study of biopolymer-degrading microorganisms in peatlands (Ivanova et al., 2016), we made an attempt to re-analyze the SSU rRNA dataset retrieved in this study with the focus on CCO287- and SBC82^T^-like bacteria. The study of Ivanova et al. (2016) assessed the substrate-induced response of peat-inhabiting microorganisms to amendments with cellulose, xylan, pectin and chitin. The 16S rRNA reads from CCO287-like and SBC82^T^-like bacteria were sorted out using an identity threshold of 99% from the sequence sets corresponding to four experimental incubations with different biopolymers and the control incubation without added substrate. This analysis revealed that the pool of 16S rRNA reads from SBC82^T^-like and CCO287-like acidobacteria significantly increased (*P*-value < 0.0001) in response to chitin availability (Supplementary Figure S1A). Some weak responses were also observed for both strains in incubations with other polymers, but these were not statistically significant. Similar tendency was observed when this analysis was performed with an identity threshold of 95% (Supplementary Figure S1B). The pool of 16S rRNA reads from *Acidisarcina*-like bacteria increased from 5% in the control to 9% in chitin-amended peat. Metatranscriptome-derived data, therefore, confirm our conclusions regarding the presence of chitinolytic potential in *Acidisarcina*-like acidobacteria.

Since strains SBC82^T^ and CCO287 were phylogenetically divergent as well as morphologically and phenotypically distinct from all earlier described Sd1 acidobacteria (Table 5), we propose to classify them as representing a novel genus and species of the family *Acidobacteriaceae*, *Acidisarcina polymorpha* gen. nov., sp. nov.

**Table 5:** Major characteristics that distinguish the genus *Acidisarcina* from the most closely related genera of the family *Acidobacteriaceae*.

Characteristic	*Acidisarcina*	*Acidicapsa*	*Occallatibacter*	*Terracidiphilus*	*Telmatobacter*	*Acidipila*	*Acidobacterium*
Motility	−	V	+	+	+	−	+
Capsule	+	+	V	+	–	+	−
Pigment	None to light pink	None to pink	None to pink	None	None	Pink	Orange
Anaerobic growth	−	−	−	−	+	−	+
pH range	4.0–7.7	2.3–7.3	3.5–8.5	3.0–6.0	3.0–7.5	3.0–8.0	3.0–6.0
pH optimum	4.8–7.0	4.0–5.0	4.0–6.0	4.0–5.0	4.5–5.0	4.0–4.5	ND
Cellulose degradation	+	−	−	+	+	−	−
Major fatty acids	13,16-Dimethyl octacosanedioic acid, iso-C15:0, C16:1ω8*c*, C16:0	13,16-Dimethyl octacosanedioic acid, iso-C15:0	13,16-Dimethyl octacosanedioic acid, iso-C15:0, iso-C17:1ω7c, iso-C17:0	iso-C15:0, C15:0, C16:1ω7c, C18:0	13,16-Dimethyl octacosanedioic acid, iso-C15:0, iso-C17:1ω8*c*	iso-C15:0, iso-C18:1ω9*c*, C16:1ω7c	13,16-Dimethyl octacosanedioic acid, iso-C15:0, C18:1ω9
G+C content (mol %)	56.8	51.7–60.0	58.5–59.9	57.3	57.6	56.3–59.5	57.2–60.8

*ND, not determined; v. variable.*

### Description of *Acidisarcina* gen. nov.

*Acidisarcina* (A.ci.di.sar.ci’na. N.L. n. *acidum*, acid; L. fem. n. *sarcina*, a package, bundle; N.L. fem. n. *Acidisarcina*, acidophilic package).

Gram-negative, non-spore-forming, highly polymorphic bacteria that occur in pairs, in sarcina-like tetrads, in clusters of 6–8 and more cells. Single cells or short chains of curved cells could also be observed occasionally. In most cases, cells occur inside saccular chambers. Colony color varies from light beige to light pink. Catalase-negative. Aerobic chemoheterotrophs. Capable of growth under micro-oxic conditions. Mild acidophiles and psychrotolerant mesophiles. Sugars are the preferred growth substrates. Capable of hydrolyzing various polysaccharides including chitin and cellulose. Menaquinone-8 is the major quinone. Major fatty acids are iso-C_15:0_, C_16:1_ω7*c*, C_16:0_ and 13,16-dimethyl octacosanedioic acid. Major polar lipids are phosphatidylethanolamine and phosphohexose; ornithine lipids can also be present. The genus belongs to the family *Acidobacteriaceae*, the order *Acidobacteriales*, the class *Acidobacteriia*. Members of this genus are typical inhabitants of acidic soils and peatlands. The type species is *Acidisarcina polymorpha*.

### Description of *Acidisarcina polymorpha* sp. nov.

*Acidisarcina* polymorpha (po.ly.mor’pha. Gr. adj. *polys*, numerous; Gr. n. *morphe*, shape. N.L. fem. adj. *polymorpha* multiform).

The description is as for the genus but with the following additional traits. Cells are 1.4–4.4 μm long and 0.9–1.5 μm wide. Carbon sources (0.05%, w/v) utilized include D-arabinose, D-fructose, D-galactose, D-glucose, D-mannose, D-xylose, melezitose, trehalose, D-lactulose, N-acetyl-D-glucosamine, maltose, melibiose, raffinose, dulcitol, and sorbitol. Utilization of lactose, D-leucrose, L-rhamnose, D-ribose, sucrose, salicin, L-sorbose, cellobiose, D-glucuronate, arbutin, inulin, mannitol, methanol, ethanol, pectin, lichenan, and laminarin is variable. Does not utilize D-fucose, pyruvate, acetate, butyrate, capronate, citrate, malate, lactate, formate, D-galacturonate, fumarate, oxalate, propionate, succinate, valerate, adonitol, arabitol, *myo*-inositol. Hydrolyze esculin, starch, and xylan but not sodium alginate, carboxymethyl-cellulose, chitosan, fucoidan, pullulan. The ability to degrade chitin and cellulose may vary between different strains. The following enzyme activities are present: alkaline and acidic phosphatase, esterase (C4), esterase lipase (C8), leucine-arylamidase, naphthol-ASBI-phosphohydrolase, β-glucosidase, valine-arylamidase, N-acetyl-β-glucosaminidase, β-galactosidase, weak activities of cystine arylamidase, β-glucuronidase and lipase (C14). Urease, trypsin, α-chymotrypsin, α-galactosidase, α-glucosidase, α-mannosidase, α-fucosidase, arginine dihydrolase and protease are absent (API ZYM test). Capable of growth at pH 4.0–7.7 (optimum pH 4.8–7.0) and at 5–36°C (optimum at 20–32°C). NaCl inhibits growth at concentrations above 1.5%. Resistant to streptomycin, kanamycin, gentamicin, tetracyclin, lincomycin, ampicillin, chloramphenicol and neomycin, but susceptible to rifampicin and novobiocin. The type strain is strain SBC82^T^ (= KCTC 82304^T^ = VKM B-3225^T^), which was isolated from acidic soil of lichen covered forested tundra in northern Russia. The DNA G+C content of the type strain is 56.8 mol%. The GenBank accession numbers for the 16S rRNA gene and the genome sequences of strain SBC82^T^ are MH396772 and CP030840-CP030844 (chromosome and four plasmids), respectively.

## Discussion

**FIGURE 5:**
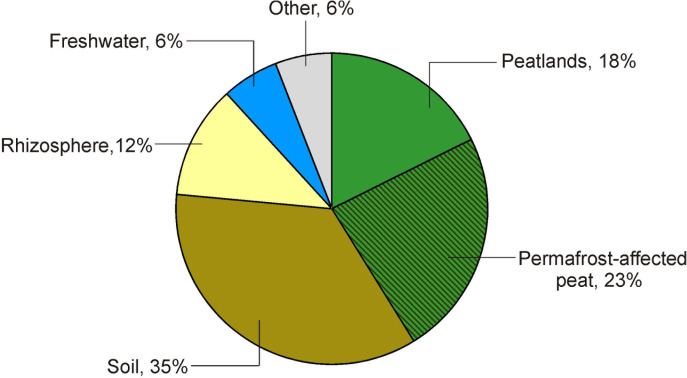
Distribution of *Acidisarcina* species in the environment. The diagram depicts origin of environmental 16S rRNA gene sequences with >95% identity to *Acidisarcina polymorpha* SBC82^T^, which were extracted from the Joint Genome Institute Integrated Microbial Genomes database (16S rRNA Public Assembled Metagenomes). More detailed information about these sequences is given in Supplementary Table S3.

The *Acidisarcina*-like bacteria appear to be common inhabitants of acidic northern soils and peatlands. In lichen-dominated forested tundra sites examined in our study, 16S rRNA gene reads from these microorganisms comprised up to 15% of all *Acidobacteria*-affiliated sequences (Figure 1b). In the boreal *Sphagnum* peat bog examined in our previous study (Ivanova et al., 2016), 16S rRNA fragments from *Acidisarcina*-like bacteria comprised ∼5% of all acidobacterial reads. Several isolates that could be classified as belonging to the genus *Acidisarcina*, strains K5 and C27 (Acc. No JF490073 and JF490072), were earlier obtained from decomposed thalli of *Cladonia* sp. collected from the ombrotrophic bog Verkhnee, Karelia (Pankratov, 2012). Acidic woodland soils are also among the habitats colonized by *Acidisarcina* species since highly similar (97–99%) 16S rRNA gene sequences (Acc. No HQ598413, HQ598652, HQ598666, HQ598998, HQ599059) were retrieved from several forest sites in Germany (Naether et al., 2012). Notably, a number of *Acidisarcina*-related 16S rRNA gene sequences were obtained from mildly acidic Hawaiian volcanic deposits (Acc. No FJ466263, FJ466272, FJ466284, FJ466322, FJ466337, FJ466355, FJ466443) (Weber and King, 2010) suggesting that these bacteria are among primary colonizers of volcanic deposits. The search for 16S rRNA sequences with >95% identity to *Acidisarcina polymorpha* SBC82^T^ in the Joint Genome Institute Integrated Microbial Genomes database (16S rRNA Public Assembled Metagenomes), revealed a total of 68 environmental sequences (>1,000 bp) (Supplementary Table S3). These 16S rRNA sequences were identified world-wide, mostly in peatlands and soils, but also in plant rhizosphere and freshwater habitats (Figure 5).

High environmental fitness and good survival of these bacteria in harsh environments could possibly be explained by a number of traits and specific adaptations. The ultrastructural peculiarity of *Acidisarcina* is that cells occur within the extracellular compartments appearing as saccular chambers (Figure 2c). Similar structures were earlier described for radiation-resistant pseudomonads (Abashina et al., 2008). These chambers could possibly serve as a structural adaptation with protective function for survival in hostile environments. The exact mechanisms remain to be investigated but the presence of cellulose synthase *bcs* operon in strain SBC82^T^ suggests its involvement in the synthesis of the chamber envelope produced by these bacteria. Similar *bcs* operons were found in *Acidobacteria* species isolated from Arctic tundra soils (Rawat et al., 2012). The genomic potential for trehalose biosynthesis could also contribute to protection of *Acidisarcina* against cold stress (Freeman et al., 2010). Protection against the cold could be also enabled by the cold-shock DNA-binding domain proteins, CspA, identified in the genome of strain SBC82^T^. Similar mechanisms of cold protection were found in the Sd1 acidobacteria isolated from Arctic tundra soils, *Granulicella tundricola*, *Terriglobus saanensis*, and *Granulicella mallensis* (Rawat et al., 2012). An extremely wide repertoire of enzymes involved in degradation of various polymers including chitin, cellulose, and xylan is apparently one of the major reasons behind high environmental relevance of these bacteria. The ability of *Acidobacteria* to degrade chitin has not received sufficient attention so far.

Much of the previously available evidence for the presence of chitinolytic capabilities in *Acidobacteria* originated from the genome analyses of these microorganisms (Ward et al., 2009; Rawat et al., 2012; Kielak et al., 2016). Our search also revealed the presence of numerous chitinase- and *N*-acetyl-beta-hexosaminidase-encoding genes in genomes and metagenomes of many Sd1 *Acidobacteria* as well as in Sd3 member ‘*Solibacter usitatus*’ and in Sd6 member *Luteitalea pratensis* (Table 6). The highest numbers of GH18 chitinases were revealed in *Granulicella mallensis* MP5ACTX8^T^ (8), *Silvibacterium bohemicum* S15^T^ (7) and *Terriglobus saanensis* SP1PR4^T^ (6). Of these bacteria, only *Silvibacterium bohemicum* S15^T^ was characterized as being capable of growth on colloidal chitin (Lladó et al., 2016). Few other taxonomic descriptions of acidobacteria also list chitin among the potential growth substrates (Foesel et al., 2013; García-Fraile et al., 2015). All of these studies, however, simply report the results of the respective growth tests on chitin as being positive and do not provide any further information. In case of *Silvibacterium bohemicum* S15^T^ and *Terracidiphilus gabretensis* S55^T^ the activity of N-acetylglucosaminidase was detected and referred as one of the proofs for growth on chitin. The most apparent reason for this lack of information is slow growth of all earlier described *Acidobacteria* on chitin. The bacterium examined in our study, strain SBC82^T^, was isolated from a habitat enriched with fungal-derived chitin, i.e., an organic soil layer underlying lichen cover. Although *Acidobacteria* are not recognized as an important component of the lichen-associated microbiome (Cernava et al., 2017), decaying lichen thalli or underlying organic layers are abundantly colonized by these bacteria (Pankratov, 2012).

**Table 6:** The presence of chitinases and *N*-acetyl-beta-hexosaminidases in *Acidobacteria.*

Acidobacterium (subdivision number)	Number of genes		
	
	GH18 family chitinase	GH19 family chitinase	GH20 family *N*-acetyl-beta-hexosaminidases
*Acidisarcina polymorpha* (1)	5	–	3
*Acidobacterium ailaaui* (1)	1	–	3
*Acidobacterium capsulatum* (1)	4	–	3
*Bryocella elongata* (1)	–	–	3
*‘Koribacter versatilis’* (1)	1	–	2
*‘Solibacter usitatus’* (3)	2	–	4
*Ca*. Sulfotelmatobacter kueseliae (1)	1	–	2
*Ca*. Sulfotelmatomonas gaucii (1)	2	–	2
*Edaphobacter aggregans* (1)	5	–	4
*Granulicella mallensis* (1)	8	–	6
*Granulicella pectinivorans* (1)	2	–	7
*Granulicella rosea* (1)	1	–	1
*Granulicella tundricola* (1)	1	–	3
*Luteitalea pratensis* (6)	1	3	–
*Silvibacterium bohemicum* (1)	7	–	3
*Terracidiphilus gabretensis* (1)	3	–	3
*Terriglobus roseus* (1)	3	1	3
*Terriglobus saanensis* (1)	5	1	3

Strains SBC82^T^ and CCO287 exemplify the situation with two members of the same species displaying difference with regard to their hydrolytic capabilities. The first bacterium was isolated from a chitin-rich habitat and grew well with chitin, while only a poor growth was observed on cellulose. The second isolate was obtained from *Sphagnum*-derived peat and displayed weak chitinolytic capabilities but demonstrated consistent growth on cellulose. These results show that hydrolytic activities may vary within members of the same species and highlight the importance of describing a bacterial species based on characterization of several independent isolates, preferably obtained from different habitats.

It has been suggested that acidobacteria become particularly important in the niches where other well-known and fast-acting bacterial decomposers, such as *Firmicutes* and *Bacteroidetes*, are absent or numerically insignificant (Pankratov et al., 2011). This is the case in ombrotrophic boreal peat bogs, lichen-dominated acidic soils and peatlands of tundra. In these habitats, *Acidobacteria* become one of the major groups of hydrolytic bacteria with *Acidisarcina* as one of the numerically abundant bacterial populations. The genome encoded ability to feed on a wide range of complex carbohydrates is one of the key traits of these acidobacteria. The ability to utilize the nitrogen-containing polymer chitin gives these bacteria an additional ecological advantage in nitrogen-depleted acidic northern wetlands and soils.

## Author Contributions

SD and SB designed the study. AI examined *Acidobacteria* diversity in soil samples and analyzed metatranscriptome-derived data. SB and TP obtained isolates of acidobacteria and performed growth experiments. NR, AB, and AM obtained, annotated, and analyzed the genome sequence. AR performed hydrolytic activity assays. JSD performed chemotaxonomic analyses. SD and NR wrote the manuscript.

## Conflict of Interest Statement

The authors declare that the research was conducted in the absence of any commercial or financial relationships that could be construed as a potential conflict of interest.

## Footnotes

1
https://github.com/jstjohn/SeqPrep

2
http://www.cbs.dtu.dk/services/SignalP/

3
http://www.compgen.org/tools/PRED-TAT/

4
http://www.cbs.dtu.dk/services/TMHMM/

5
www.graphpad.com
