# Corrigendum: Hydrolytic Capabilities as a Key to Environmental Success: Chitinolytic and Cellulolytic *Acidobacteria* From Acidic Sub-arctic Soils and Boreal Peatlands

**DOI:** 10.3389/fmicb.2022.856396

**Published:** 2022-02-24

**Authors:** Svetlana E. Belova, Nikolai V. Ravin, Timofey A. Pankratov, Andrey L. Rakitin, Anastasia A. Ivanova, Alexey V. Beletsky, Andrey V. Mardanov, Jaap S. Sinninghe Damsté, Svetlana N. Dedysh

**Affiliations:** ^1^Winogradsky Institute of Microbiology, Research Center of Biotechnology of the Russian Academy of Sciences, Moscow, Russia; ^2^Institute of Bioengineering, Research Center of Biotechnology of the Russian Academy of Sciences, Moscow, Russia; ^3^M.V. Lomonosov Moscow State University, Moscow, Russia; ^4^Department of Marine Microbiology and Biogeochemistry, Royal Netherlands Institute for Sea Research, Utrecht University, Den Burg, Netherlands; ^5^Geochemistry, Department of Earth Sciences, Faculty of Geosciences, Utrecht University, Utrecht, Netherlands

**Keywords:** *Acidobacteria*, *Acidisarcina*, lichen-covered tundra, genome annotation, chitinase, chitinolytic ability

The description of the novel genus and species, *Acidisarcina polymorpha* gen. nov., sp. nov., published in the original article did not include the information about the type species of the newly proposed genus as well as the information regarding the designations allotted to the type strain of the novel species by two different culture collections. The published protologue of *Acidisarcina polymorpha*, therefore, does not fulfill the requirements of Rule 30 (3) b of the International Code of Nomenclature of Prokaryotes. This Corrigendum was prepared in order to correct this mistake and to present missing information, which is required for valid publication of the proposed new names.

A correction has been made to the **Results**, subsection **Description of** ***Acidisarcina*** **gen. nov.:**

**“Description of** ***Acidisarcina*** **gen. nov**.

*Acidisarcina* (A.ci.di.sar.ci'na. N.L. n. *acidum*, acid; L. fem. n. *sarcina*, a package, bundle; N.L. fem. n. *Acidisarcina*, acidophilic package).

Gram-negative, non-spore-forming, highly polymorphic bacteria that occur in pairs, in sarcina-like tetrads, in clusters of 6–8 and more cells. Single cells or short chains of curved cells could also be observed occasionally. In most cases, cells occur inside saccular chambers. Colony color varies from light beige to light pink. Catalase-negative. Aerobic chemoheterotrophs. Capable of growth under micro-oxic conditions. Mild acidophiles and psychrotolerant mesophiles. Sugars are the preferred growth substrates. Capable of hydrolyzing various polysaccharides including chitin and cellulose. Menaquinone-8 is the major quinone. Major fatty acids are iso-C_15:0_, C_16:1_ω7*c*, C_16:0_ and 13,16-dimethyl octacosanedioic acid. Major polar lipids are phosphatidylethanolamine and phosphohexose; ornithine lipids can also be present. The genus belongs to the family *Acidobacteriaceae*, the order *Acidobacteriales*, the class *Acidobacteriia*. Members of this genus are typical inhabitants of acidic soils and peatlands. The type species is *Acidisarcina polymorpha*.”

Additionally, a correction has been made to the **Results**, subsection **Description of** ***Acidisarcina polymorpha*** **sp. nov.:**

**“Description of** ***Acidisarcina polymorpha*** **sp. nov**.

*Acidisarcina polymorpha* (po.ly.mor'pha. Gr. adj. *polys*, numerous; Gr. n. *morphe*, shape. N.L. fem. adj. *polymorpha* multiform).

The description is as for the genus but with the following additional traits. Cells are 1.4–4.4 μm long and 0.9–1.5 μm wide. Carbon sources (0.05%, w/v) utilized include D-arabinose, D-fructose, D-galactose, D-glucose, D-mannose, D-xylose, melezitose, trehalose, D-lactulose, N-acetyl-D-glucosamine, maltose, melibiose, raffinose, dulcitol, and sorbitol. Utilization of lactose, D-leucrose, L-rhamnose, D-ribose, sucrose, salicin, L-sorbose, cellobiose, D-glucuronate, arbutin, inulin, mannitol, methanol, ethanol, pectin, lichenan, and laminarin is variable. Does not utilize D-fucose, pyruvate, acetate, butyrate, capronate, citrate, malate, lactate, formate, D-galacturonate, fumarate, oxalate, propionate, succinate, valerate, adonitol, arabitol, *myo*-inositol. Hydrolyze esculin, starch, and xylan but not sodium alginate, carboxymethyl-cellulose, chitosan, fucoidan, pullulan. The ability to degrade chitin and cellulose may vary between different strains. The following enzyme activities are present: alkaline and acidic phosphatase, esterase (C4), esterase lipase (C8), leucine-arylamidase, naphthol-ASBI-phosphohydrolase, β-glucosidase, valine-arylamidase, N-acetyl-β-glucosaminidase, β-galactosidase, weak activities of cystine arylamidase, β-glucuronidase and lipase (C14). Urease, trypsin, α-chymotrypsin, α-galactosidase, α-glucosidase, α-mannosidase, α-fucosidase, arginine dihydrolase and protease are absent (API ZYM test). Capable of growth at pH 4.0–7.7 (optimum pH 4.8–7.0) and at 5–36°C (optimum at 20–32°C). NaCl inhibits growth at concentrations above 1.5%. Resistant to streptomycin, kanamycin, gentamicin, tetracyclin, lincomycin, ampicillin, chloramphenicol and neomycin, but susceptible to rifampicin and novobiocin. The type strain is strain SBC82^T^ (= KCTC 82304 ^T^ = VKM B-3225^T^), which was isolated from acidic soil of lichen covered forested tundra in northern Russia. The DNA G+C content of the type strain is 56.8 mol%. The GenBank accession numbers for the 16S rRNA gene and the genome sequences of strain SBC82^T^ are MH396772 and CP030840-CP030844 (chromosome and four plasmids), respectively.”

The authors apologize for this error and state that this does not change the scientific conclusions of the article in any way. The original article has been updated.

## Publisher's Note

All claims expressed in this article are solely those of the authors and do not necessarily represent those of their affiliated organizations, or those of the publisher, the editors and the reviewers. Any product that may be evaluated in this article, or claim that may be made by its manufacturer, is not guaranteed or endorsed by the publisher.

