# Supplementary material for: Hydrolytic Capabilities as a Key to Environmental Success: Chitinolytic and Cellulolytic Acidobacteria From Acidic Sub-arctic Soils and Boreal Peatlands
Source: Front Microbiol. 2018 Nov 19;9:2775. doi: 10.3389/fmicb.2018.02775 (PMC6252331; doi:10.3389/fmicb.2018.02775)
Supplement: Supplementary file 1 [file Data_Sheet_1.PDF]

SUPPORTING INFORMATION

Hydrolytic capabilities as a key to environmental success: chitinolytic and cellulolytic *Acidobacteria* from acidic sub-arctic soils and boreal peatlands

Svetlana E. Belova[1], Nikolai V. Ravin[2], Timofey A. Pankratov[3], Andrey L. Rakitin[2], Anastasia A. Ivanova[1], Alexey V. Beletsky[2], Andrey V. Mardanov[2], Jaap S. Sinninghe Damsté[4,5], and Svetlana N. Dedysh[1*]

[1]Winogradsky Institute of Microbiology, Research Center of Biotechnology of the Russian Academy of Sciences, Moscow 119071, Russia;

[2]Institute of Bioengineering, Research Center of Biotechnology of the Russian Academy of Sciences, Moscow 119071, Russia;

[3]M.V. Lomonosov Moscow State University, GSP-1, Moscow 119234, Russia.

[4]NIOZ Royal Netherlands Institute for Sea Research, Department of Marine Microbiology and Biogeochemistry, and Utrecht University, PO Box 59, 1790 AB Den Burg, the Netherlands;

[5]Utrecht University, Faculty of Geosciences, Department of Earth Sciences, Geochemistry, Utrecht, the Netherlands.

Running title: new chitinolytic acidobacteria.

***Correspondence**: Dr. Svetlana N. Dedysh, Winogradsky Institute of Microbiology, Research Center of Biotechnology of the Russian Academy of Sciences, Moscow 119071, Russia; email: dedysh@mail.ru, phone +7(499) 1350591; fax +7(499) 1356530.

CONTENT:

Supplementary Table S1. Genes of *Acidisarcina polymorpha* SBC82^T, involved in metabolic pathways discussed in the text.

Supplementary Table S2. Genes of *Acidisarcina polymorpha* SBC82^T, involved in hydrolysis of polysaccharides, discussed in the text.

Supplementary Table S3. Environmental distribution of *Acidisarcina* species.

Supplementary Figure S1. The relative abundance values represent average values calculated by relating the number of reads assigned to *A. polymorpha* strains SBC82^T (black bars) and CCO287 (grey bars) (blast sequence identity threshold of 99%) (**A**) or to *Acidisarcina*-like bacteria (blast sequence identity threshold of 95%) (**B**) to the total number of SSU rRNA reads retrieved from four experimental incubations of peat samples amended with different biopolymers and the control incubation without added substrate in the study of Ivanova et al. (2016).

Supplementary Table S1. Genes of *Acidisarcina polymorpha* SBC82^{T}, involved in metabolic pathways discussed in the text.

Gene ID (ACPOL_)*	Predicted protein
	**Embden-Meyerhof pathway**
2769, 3582, 5348, 7071	glucokinase
6081	glucose-6-phosphate isomerase
5611	6-phosphofructokinase
3242	fructose bisphosphate aldolase
20	triose phosphate isomerase
14	glyceraldehyde-3 phosphate dehydrogenase
15	3-phosphoglycerate kinase
167, 1978. 5096	phosphoglycerate mutase
165	enolase
4449	pyruvate kinase
1528	fructose-1,6-bisphosphatase
	**Pentose phosphate pathway**
5345, 5346, 5687	Glucose-6-phosphate 1-dehydrogenase
2243, 2785, 5347	6-phosphogluconolactonase
0706, 1151, 1804	6-phosphogluconate dehydrogenase
6088	ribulose phosphate 3-epimerase
4876, 5343	ribose 5-phosphate isomerase
5342	transketolase
3877, 6835	transaldolase
	**Pyruvate metabolism**
5879	Pyruvate-flavodoxin oxidoreductase
5166	Phosphoenolpyruvate carboxykinase
2325	Phosphoenolpyruvate carboxylase
5668	Phosphate acetyltransferase
1372, 1981	Acetate kinase
0564, 1483, 2834, 2835, 3673, 3774, 4581, 5000, 6453, 6454, 6468	Aldehyde dehydrogenase
0665, 0742, 0743, 1148, 1479, 2862, 2864, 2865, 3029, 3148, 3975, 4029, 4574, 4582, 4667, 5126, 5223, 5956, 6557, 6560, 7182	Alcohol dehydrogenase
	**Tricarboxylic acid cycle and related enzymes**
1815	Citrate synthase
2395	aconitate hydratase
1167, 6022	Isocitrate dehydrogenase
6663	2-oxoglutarate oxidoreductase, alpha subunit
6661,	2-oxoglutarate oxidoreductase, beta subunit
1190, 3609	succinyl-CoA synthetase, alpha subunit
0885, 1543, 3610, 7108	succinyl-CoA synthetase, beta subunit
6016	Succinate dehydrogenase iron-sulfur protein
6015	Succinate dehydrogenase flavoprotein subunit
6014	Succinate dehydrogenase cytochrome b558 subunit

0381, 0730, 1787, 1858	Fumarate hydratase
0714, 6021	Malate dehydrogenase
0732, 1859	Malic enzyme
	**Glycogen and trehalose metabolism**
3623	glucose-1-phosphate adenylyltransferase
4979, 5731	glycogen synthase
5901	glycogen branching enzyme
0857, 3074, 5692	trehalose synthase
5754	maltooligosyl trehalose synthase
1892, 2499, 6240	maltooligosyl trehalose trehalohydrolase
	**Respiratory chain**
5835	NADH-ubiquinone oxidoreductase chain A
0226	NADH-ubiquinone oxidoreductase chain B
5836	NADH-ubiquinone oxidoreductase chain C
5837	NADH-ubiquinone oxidoreductase chain D
5839	NADH-ubiquinone oxidoreductase chain E
5840	NADH-ubiquinone oxidoreductase chain F
5843	NADH-ubiquinone oxidoreductase chain G
5844	NADH-ubiquinone oxidoreductase chain H
5116	NADH-ubiquinone oxidoreductase chain I
5845	NADH-ubiquinone oxidoreductase chain J
5846	NADH-ubiquinone oxidoreductase chain K
5847	NADH-ubiquinone oxidoreductase chain L
5848	NADH-ubiquinone oxidoreductase chain M
5849	NADH-ubiquinone oxidoreductase chain N
5784	ubiquinol--cytochrome c reductase, cytochrome B subunit
5785	ubiquinol-cytochrome c reductase iron-sulfur subunit
5786	cytochrome c family protein
1645	SCO1/SenC family protein
1644, 5671, 5790	Cytochrome c oxidase polypeptide II
1643, 5672, 5789	Cytochrome c oxidase polypeptide I
1642, 5673, 5788	Cytochrome c oxidase polypeptide III
1641, 5787	Cytochrome c oxidase polypeptide IV
2269	Heme A synthase, CtaA
5951	Heme O synthase, protoheme IX farnesyltransferase CtaB
5243	F_0F_1-type ATPase, F0 sector subunit a
2034	F_0F_1-type ATPase, F0 sector subunit b
2035	F_0F_1-type ATPase, F0 sector subunit b’
5244	F_0F_1-type ATPase, F0 sector subunit c
2032	F_0F_1-type ATPase, alpha chain
2030	F_0F_1-type ATPase, beta chain
2031	F_0F_1-type ATPase, gamma chain
2033	F_0F_1-type ATPase, delta chain
2029	F_0F_1-type ATPase, epsilon chain
4124	Pyrophosphate-energized proton pump
	**Hydrogenases**
5871	Group 3d [NiFe] NAD-reducing hydrogenase subunit HoxE
5872	Group 3d [NiFe] NAD-reducing hydrogenase subunit HoxF
5873	Group 3d [NiFe] NAD-reducing hydrogenase subunit HoxU
5874	Group 3d [NiFe] NAD-reducing hydrogenase subunit HoxY

5875	Group 3d [NiFe] NAD-reducing hydrogenase subunit HoxH
0992	Group 1h [NiFe] uptake hydrogenase large subunit
0993	Group 1h [NiFe] uptake hydrogenase small subunit
	**Anaerobic respiration**
0081, 4671	Molybdopterin oxidoreductase, catalytic subunit
0082, 4670	Molybdopterin oxidoreductase, cytochrome B subunit (membrane anchoring protein)
1652	Molybdopterin oxidoreductase, chaperone protein HtpG
1651	Molybdopterin oxidoreductase, fused catalytic and iron-sulfur binding subunits
1650	Molybdopterin oxidoreductase, NrfD-like subunit
	**Lactate utilization lutABC operon**
2407, 4357	L-lactate utilization protein LutA/YkgE
2406, 4358	L-lactate utilization protein LutB/YkgF
2405, 4359	L-lactate utilization protein LutC
	**Cellulose synthase**
3660	Cellulase (glycosyl hydrolase family GH5)
3661	Bacterial cellulose synthase protein BcsQ
3662	Bacterial cellulose synthase, fused subunits BcsA and BcsB
3663, 3664	Bacterial cellulose synthase subunit BcsC

* Gene ID (locus_tag) according to GenBank CP030840

Supplementary Table S2. Genes of *Acidisarcina polymorpha* SBC82^{T}, involved in hydrolysis of polysaccharides, discussed in the text.

Gene ID	Signal peptide	Predicted protein
		**Hydrolysis of chitin**
6017	+	Chitinase GH18 family
1623	+	Chitinase GH18 family
3848	-	Chitinase GH18 family
2565	+	Chitinase GH18 family
5649	+	Chitinase GH18 family
6023	+	N-acetyl-beta-hexosaminidase, GH20 family
2385	+	N-acetyl-beta-hexosaminidase, GH20 family
4892	+	N-acetyl-beta-hexosaminidase, GH20 family
985	-	Beta-glucosidase/ beta-hexosaminidase, GH3 family
5806	-	Beta-glucosidase/ beta-hexosaminidase, GH3 family
		**Hydrolysis of starch**
5097	+	Alpha-amylase / oligo-1,6-glucosidase
1665	+	Alpha-amylase / oligo-1,6-glucosidase
4191	+	Alpha-amylase
489	+	Amylo-alpha-1,6-glucosidase
5341	-	Glucoamylase (glucan-1,4-alpha-glucosidase), GH15 family
5693	-	Alpha-amylase
1655	-	Alpha-amylase /alpha-mannosidase, GH57 family
1435	-	Alpha-amylase
2893	-	Alpha-amylase / oligo-1,6-glucosidase
		**Hydrolysis of xylan**
4002	+	Endo-1,4-beta-xylanase, GH10 family
3367	+	Endo-1,4-beta-xylanase, GH10 family
4467	+	Beta-xylosidase, GH39 family
2922	+ (TAT)	Beta-xylosidase, GH43 family
3600	+	Beta-xylosidase, GH39 family
		**Hydrolysis of cellulose**
1013	+	Cellulase, GH5 family
3508	+ (TAT)	Cellulase, GH5 family
6425	+	Cellulase, GH5 family
2075	+	Endoglucanase, GH44 family
1431	+	Beta-glucosidase, GH1 family
2136	+	Beta-glucosidase, GH3 family
4613	+	Beta-glucosidase, GH3 family
1722	+	Beta-glucosidase, GH3 family
4613	+	Beta-glucosidase, GH3 family
2003	+	Beta-glucosidase, GH3 family
		**Other enzymes**
3286	+	Laminarinase/ endo-1,3-beta-D-glucosidase, GH16 family
7063	+	Alpha-mannosidase, GH38 family
3284	+ (TAT)	glycosyl hydrolase GH64 family

* TAT, twin-arginine signal peptides.

Supplementary Table S3. Environmental distribution of *Acidisarcina* species. The table contains data on 16S rRNA gene sequences (>1000 bp) from the IMG database with > 95% identity to the 16S rRNA of *Acidisarcina polymorpha* SBC82.

**Gene ID**	**Genome ID**	**Metagenome Name**	**Subject Length**	**Identities**
Ga0078972_1000045272	3300005573	Hot spring, Yellowstone National Park, USA	1504	96%
Ga0224518_10003444	3300022824	Peat soil, Stordalen Mire	1506	96%
Ga0224533_10009373	3300022526	Peat soil, Stordalen Mire, Sweden	1506	96%
Ga0213871_100056461	3300021441	Rhizosphere, Vellozia epidendroides, National Park of Serra do Cipo, Brazil	1504	96%
Ga0213872_100309541	3300021361	Rhizosphere, Vellozia epidendroides, National Park of Serra do Cipo, Brazil	1504	96%
Ga0164296_100195324	3300013093	Dystrophic water lake, Trout Bog Lake, Wisconsin, USA	1504	95%
Ga0248169_10325217	3300022602	Dystrophic water lake, Trout Bog Lake, Wisconsin, USA	1504	95%
Ga0164297_1000687410	3300013094	Dystrophic water lake, Trout Bog Lake, Wisconsin, USA	1504	95%
Ga0224557_10063916	3300023101	Peat soil, Stordalen Mire, Sweden	1506	95%
Ga0182028_13531621	3300019788	Permafrost Stordalen Mire, Sweden	1483	95%
Ga0224553_100045514	3300022875	Peat soil, Stordalen Mire, Sweden	1504	95%
JGI12675J13321_1001761	3300001140	Forest soil, Davy Crockett National Forest, Texas, USA	1380	96%
Ga0224530_10001089	3300023228	Peat soil, Stordalen Mire, Sweden	1506	95%
Ga0224529_10002024	3300023247	Peat soil, Stordalen Mire, Sweden	1506	95%

TB_PC08_64DRAFT_10030926	3300000232	Subsurface biofilms in sulfidic aquifier in Frasassi Gorge, Italy,	1382	96%
Ga0224531_100011934	3300022849	Peat soil, Stordalen Mire, Sweden	1506	95%
Ga0182031_10264715	3300019787	Peat soil, Stordalen Mire, Sweden	1456	95%
Ga0182028_15463881	3300019788	Permafrost, Stordalen Mire, Sweden	1403	96%
Ga0182022_13592112	3300019785	Permafrost, Stordalen Mire, Sweden	1433	95%
JGI12688J13320_1000891	3300001134	Forest soil, Davy Crockett National Forest, Texas, USA	1382	95%
TBL_comb47_HYPODRAFT_100578401	3300000553	Dystrophic water lake, Trout Bog Lake, Wisconsin, USA	1382	95%
Ga0182028_13072741	3300019788	Permafrost, Stordalen Mire, Sweden	1387	95%
JGI994J12354_100588511	3300000839	Passalidae beetle gut microbial communities, Costa Rica	1382	95%
IMNBL3_100588511	3300000114	Passalidae beetle gut microbial communities, Costa Rica	1382	95%
Ga0182031_13488051	3300019787	Permafrost, Stordalen Mire, Sweden	1418	95%
JGI12645J13327_1002122	3300001144	Forest soil ,Algoma, Ontario, Canada	1384	95%
JGI12491J13347_1008961	3300001132	Tropical forest soil, Luquillo Experimental Forest, Puerto Rico	1349	95%
Ga0224549_10118941	3300022840	Peat soil, Stordalen Mire, Sweden	1254	97%
Ga0182025_13582531	3300019786	Permafrost, Stordalen Mire, Sweden	1389	95%
Ga0182031_15372501	3300019787	Permafrost, Stordalen Mire, Sweden	1358	96%

Ga0224528_10208241	3300022861	Peat soil, Stordalen Mire, Sweden	1380	95%
Ga0182031_14424671	3300019787	Permafrost, Stordalen Mire, Sweden	1315	96%
Ga0137414_11798031	3300015051	Vadose zone soil, Angelo Coast Range Reserve, California, USA	1350	95%
JGI12669J13542_1004121	3300001173	Forest soil, Algoma, Ontario, Canada	1298	95%
Ga0182025_13328224	3300019786	Permafrost, Stordalen Mire, Sweden	1280	96%
Ga0213878_100469901	3300021444	Vellozia epidendroides bulk soil, National Park of Serra do Cipo, Brazil	1229	97%
Ga0224545_10074291	3300022881	Peat soil, Stordalen Mire, Sweden	1239	96%
Ga0182031_14532101	3300019787	Permafrost, Stordalen Mire, Sweden	1227	95%
Ga0070739_101554891	3300005532	Surface soil, recovering from an underground coalmine fire, Centralia Pennsylvania	1198	97%
Ga0224534_100055224	3300022524	Peat soil, Stordalen Mire, Sweden	1194	97%
Ga0179590_10406451	3300020140	Vadose zone soil , Angelo Coast Range Reserve, California, USA	1164	97%
Ga0137390_104547171	3300012363	Vadose zone soil , Angelo Coast Range Reserve, California, USA	1227	95%
Ga0193707_10587411	3300019881	Soil, a riparian zone of the East river system, Colorado, USA	1190	96%
Ga0210397_100451155	3300021403	Forest soil, Barre Woods Harvard Forest, Massachusetts, USA	1229	95%
Ga0137376_102186512	3300012208	Vadose zone soil , Angelo Coast Range Reserve, California, USA	1228	95%

Ga0099830_101312171	3300009088	Vadose zone soil , Angelo Coast Range Reserve, California, USA	1221	95%
Ga0182031_14084271	3300019787	Permafrost, Stordalen Mire, Sweden	1197	95%
Ga0224515_10103071	3300022830	Peat soil, Stordalen Mire, Sweden	1224	95%
Ga0068859_1003778931	3300005617	Switchgrass rhizosphere, Kellogg Biological Station, Michigan, USA	1221	95%
Ga0097620_1003778621	3300006931	Switchgrass rhizosphere, Kellogg Biological Station, Michigan, USA	1221	95%
Ga0066704_101624301	3300005557	Grasslands soil, Angelo Coastal Reserve, California, USA	1223	95%
Ga0193709_10172683	3300021411	Soil, a riparian zone of the East river system, Colorado, USA	1223	95%
JGI12696J13243_1001881	3300001079	Forest soil ,Davy Crockett National Forest, Texas, USA	1136	96%
JGI12631J13338_10113971	3300001131	Forest soil ,Thunder Bay, Ontario, Canada	1042	98%
Ga0164304_101230561	3300012986	Soil amended with pyrogenic organic matter, NY, USA	1207	95%
Ga0068865_1001272814	3300006881	Miscanthus rhizosphere, Kellogg Biological Station, Michigan, USA	1181	95%
Ga0182028_11994362	3300019788	Permafrost, Stordalen Mire, Sweden	1136	95%
Ga0182031_12343112	3300019787	Permafrost, Stordalen Mire, Sweden	1091	96%
Ga0182031_12130171	3300019787	Permafrost, Stordalen Mire, Sweden	1121	95%
Ga0070699_1005241441	3300005518	Corn, switchgrass and miscanthus rhizosphere , Kellogg Biological Station, Michigan, USA	1077	95%

Ga0182031_14601521	3300019787	Permafrost, Stordalen Mire, Sweden	1102	95%
Ga0134124_102347393	3300010397	Terrestrial soil without Nitrogen fertilizer, Kellogg Biological Station, Michigan, USA	1097	96%
JGI12057J13267_1001271	3300001381	Forest soil ,El Dorado National Forest, California, USA	1088	95%
Ga0182032_104635451	3300016357	Lab enrichment of tropical soil, Luquillo Experimental Forest, Puerto Rico	1036	96%
Ga0164309_103742211	3300012984	Soil amended with fresh organic matter, NY, USA	1053	95%
Ga0068862_1006263501	3300005844	Switchgrass rhizosphere, Kellogg Biological Station, Michigan, USA	1035	95%
Ga0073909_101354281	3300005526	Surface soil , recovering from an underground coalmine fire, Centralia Pennsylvania	1012	95%
Ga0105246_105286681	3300011119	Miscanthus rhizosphere , Kellogg Biological Station, Michigan, USA	1007	95%

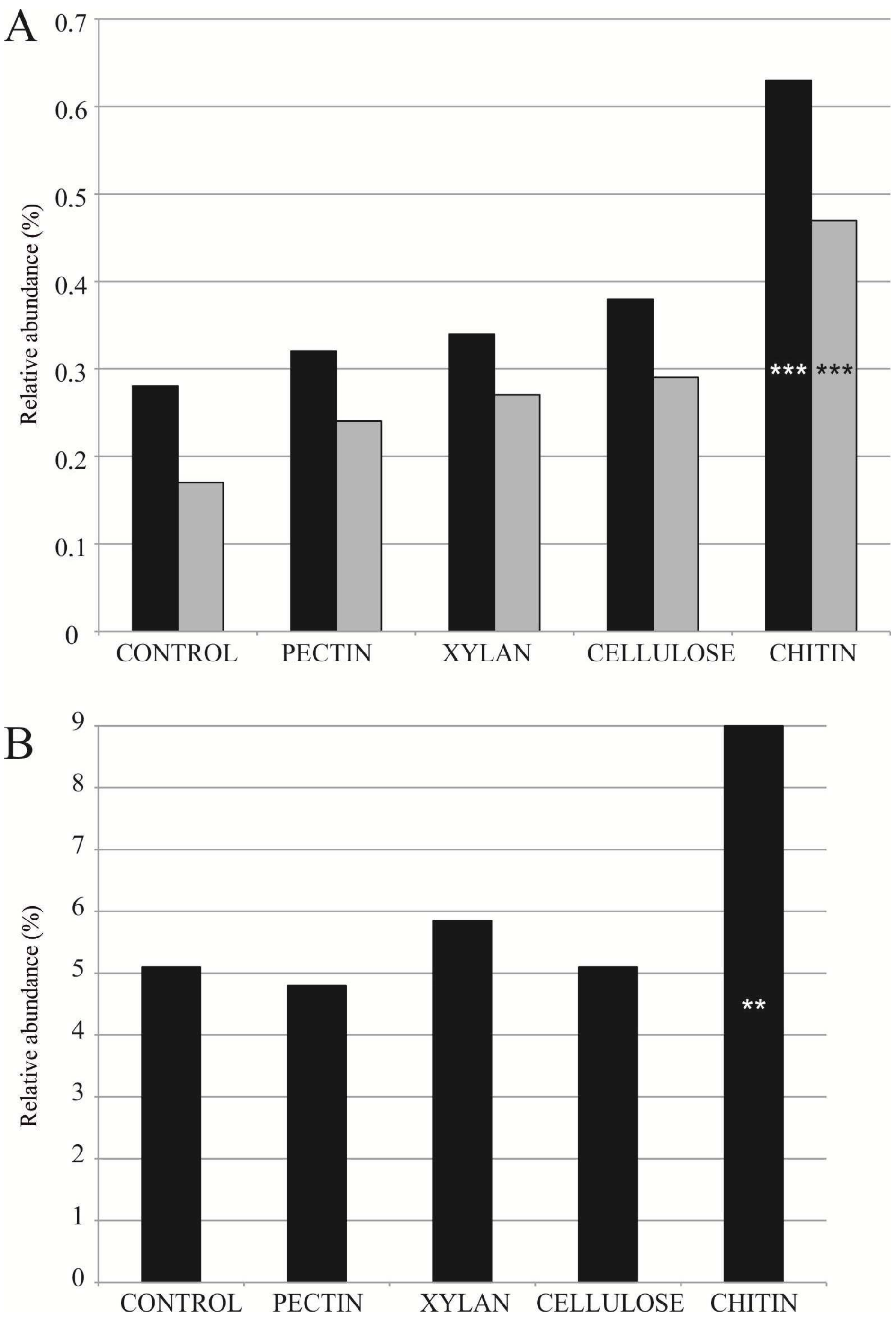

Supplementary Figure S1. The relative abundance values represent average values calculated by relating the number of reads assigned to *A. polymorpha* strains SBC82^T (black bars) and CCO287 (grey bars) (blast sequence identity threshold of 99%) (**A**) or to *Acidisarcina*-like bacteria (blast sequence identity threshold of 95%) (**B**) to the total number of SSU rRNA reads retrieved from four experimental incubations of peat samples amended with different biopolymers and the control incubation without added substrate in the study of Ivanova et al. (2016). Significant difference in ribo-tag abundances between control and biopolymer-amended samples is indicated by the asterisks: ***P*-value confidence level <0.001 and *** *P*-value confidence level <0.0001.

Reference: Ivanova, A. A., Wegner, C. E., Kim, Y., Liesack, W., and Dedysh, S. N. (2016). Identification of microbial populations driving biopolymer degradation in acidic peatlands by metatranscriptomic analysis. *Mol. Ecol.* 25, 4818–4835. doi:10.1111/mec.13806.
